# Genome-wide association analysis of type 2 diabetes in the EPIC-InterAct study

**DOI:** 10.1038/s41597-020-00716-7

**Published:** 2020-11-13

**Authors:** Lina Cai, Eleanor Wheeler, Nicola D. Kerrison, Jian’an Luan, Panos Deloukas, Paul W. Franks, Pilar Amiano, Eva Ardanaz, Catalina Bonet, Guy Fagherazzi, Leif C. Groop, Rudolf Kaaks, José María Huerta, Giovanna Masala, Peter M. Nilsson, Kim Overvad, Valeria Pala, Salvatore Panico, Miguel Rodriguez-Barranco, Olov Rolandsson, Carlotta Sacerdote, Matthias B. Schulze, Annemieke M. W. Spijkerman, Anne Tjonneland, Rosario Tumino, Yvonne T. van der Schouw, Stephen J. Sharp, Nita G. Forouhi, Elio Riboli, Mark I. McCarthy, Inês Barroso, Claudia Langenberg, Nicholas J. Wareham

**Affiliations:** 1 MRC Epidemiology Unit, University of Cambridge, Cambridge, United Kingdom; 2 Clinical Pharmacology Centre, Queen Mary University of London, London, United Kingdom; 3 Department of Clinical Sciences, Clinical Research Center, Skåne University Hospital, Lund University, 20502 Malmö, Sweden; 4 Department of Public Health and Clinical Medicine, Umeå University, 90187 Umeå, Sweden; 5 Ministry of Health of the Basque Government, Public Health Division of Gipuzkoa, Biodonostia Health Research Institute, Donostia-San Sebastian, Spain; 6 Navarra Public Health Institute, Pamplona, Spain; 7 IdiSNA, Navarra Institute for Health Research, Pamplona, Spain; 8 Centro de Investigación Biomédica en Red de Epidemiología y Salud Pública (CIBERESP), Madrid, Spain; 9 Unit of Nutrition and Cancer, Catalan Institute of Oncology - ICO, Nutrition and Cancer Group, Bellvitge Biomedical Research Institute - IDIBELL, L’Hospitalet de Llobregat, Barcelona, 08908 Spain; 10 Digital Epidemiology and e-Health Research Hub, Department of Population Health, Luxembourg Institute of Health, 1A-B, rue Thomas Edison, L-1445 Strassen, Luxembourg; 11 Center of Epidemiology and Population Health UMR 1018, Inserm, Paris South - Paris Saclay University, Gustave Roussy Institute, Villejuif, France; 12 Division of Cancer Epidemiology, German Cancer Research Center (DKFZ), Im Neuenheimer Feld 581, 69120 Heidelberg, Germany; 13 Department of Epidemiology, Murcia Regional Health Council, IMIB-Arrixaca, Murcia, Spain; 14 Cancer Risk Factors and Life-Style Epidemiology Unit, Institute for Cancer Research, Prevention and Clinical Network - ISPRO, Florence, Italy; 15 Department of Public Health, Aarhus University, Bartholins Allé 2, DK-8000 Aarhus C, Denmark; 16 Department of Cardiology, Aalborg University Hospital, Sdr. Skovvej 15, DK-9000 Aalborg, Denmark; 17 Epidemiology and Prevention Unit, Fondazione IRCCS Istituto Nazionale dei Tumori di Milano, Milan, Italy; 18 Dipartimento di Medicina Clinica e Chirurgia, Federico II University, via Pansini 5, 80131 Naples, Italy; 19 Escuela Andaluza de Salud Pública (EASP), Granada, Spain; 20 Instituto de Investigación Biosanitaria ibs.Granada, Granada, Spain; 21 Unit of Cancer Epidemiology, Città della Salute e della Scienza University-Hospital and Center for Cancer Prevention (CPO), Turin, Italy; 22 Department of Molecular Epidemiology, German Institute of Human Nutrition Potsdam-Rehbruecke, Nuthetal, Germany; 23 German Center for Diabetes Research (DZD), Neuherberg, Germany; 24 Institute of Nutrition Science, University of Potsdam, Nuthetal, Germany; 25 National Institute for Public Health and the Environment (RIVM), PO Box 1, 3720 BA Bilthoven, The Netherlands; 26 Danish Cancer Society Research Center, Strandboulevarden 49, 2100 Copenhagen, Denmark; 27 Cancer Registry and Histopathology Department, Azienda Sanitaria Provinciale No 7, Piazza Igea Nr 1, 97100 Ragusa, Italy; 28 Associazone Iblea per la Ricerca Epidemiologica -Organizazione Non Lucrativa di Utilità Sociale, Piazza Amcione No 2, 97100 Ragusa, Italy; 29 Julius Center for Health Sciences and Primary Care, University Medical Center Utrecht, Utrecht University, 3584 CG Utrecht, the Netherlands; 30 School of Public Health, Imperial College London, London, United Kingdom; 31 Wellcome Centre for Human Genetics, University of Oxford, Oxford, United Kingdom; 32 Oxford Centre for Diabetes, University of Oxford, Oxford, United Kingdom; 33 Oxford NIHR Biomedical Research Centre, Oxford University Hospitals NHS Foundation Trust, John Radcliffe Hospital, Oxford, United Kingdom; 34 University of Exeter Medical School, Exeter, United Kingdom; 35 Present Address: 1 DNA Way, South San Francisco, CA 94080 USA

**Keywords:** Epidemiology, Genetics research, Type 2 diabetes

## Abstract

Type 2 diabetes (T2D) is a global public health challenge. Whilst the advent of genome-wide association studies has identified >400 genetic variants associated with T2D, our understanding of its biological mechanisms and translational insights is still limited. The EPIC-InterAct project, centred in 8 countries in the European Prospective Investigations into Cancer and Nutrition study, is one of the largest prospective studies of T2D. Established as a nested case-cohort study to investigate the interplay between genetic and lifestyle behavioural factors on the risk of T2D, a total of 12,403 individuals were identified as incident T2D cases, and a representative sub-cohort of 16,154 individuals was selected from a larger cohort of 340,234 participants with a follow-up time of 3.99 million person-years. We describe the results from a genome-wide association analysis between more than 8.9 million SNPs and T2D risk among 22,326 individuals (9,978 cases and 12,348 non-cases) from the EPIC-InterAct study. The summary statistics to be shared provide a valuable resource to facilitate further investigations into the genetics of T2D.

## Abstract (summary)

**Table:** 

Measurement(s)	type 2 diabetes mellitus
Technology Type(s)	case-cohort study • genome wide association study
Factor Type(s)	genotype dosage • genetic principal components • study centre • Age • Sex
Sample Characteristic - Organism	Homo sapiens
Sample Characteristic - Location	Europe

Machine-accessible metadata file describing the reported data: 10.6084/m9.figshare.12981821

## Background & Summary

Diabetes is one of the fastest-growing health challenges of the 21^st^ century. The most common form of diabetes, type 2 diabetes (T2D), is a complex multifactorial disease which can lead to further severe health consequences such as cardiovascular diseases and premature death. In 2019, 463 million people worldwide were living with diabetes according to the International Diabetes Federation, and this number is expected to rise to 700 million by 2045^1^. Genome-wide association studies (GWAS) have made considerable progress in identifying genetic risk factors and in providing evidence for more in-depth understanding of the biological and pathological pathways underlying T2D. A recent study performed a meta-analysis of T2D across 32 GWAS of European ancestry participants and identified 243 genome-wide significant loci (403 distinct genetic variants) associated with T2D risk^2^. The summary statistics from this meta-analysis are publicly available; however, the GWAS results for each participating study, including EPIC-InterAct, cannot be acquired easily.

To date, a growing body of comprehensive methods has been developed for downstream analyses of GWAS. Sharing of summary statistics can help enable these analyses, for example, by providing researchers with a more convenient way to look-up genetic association effect estimates to conduct causal inference analyses using methods such as two-sample Mendelian Randomization which assumes samples are non-overlapping^3,4^. In addition, sharing GWAS results can help researchers to further their understanding of the shared genetic basis of T2D with other traits of interest, to perform fine-mapping to pinpoint the causal genetic variants or identify genetic loci shared with other risk factors and disease outcomes. Therefore, the aim of this current work was to provide a reference dataset for researchers to utilize in order to conduct further genetic analyses, generate hypotheses and improve understanding of the aetiology, the biological pathways and mechanisms of T2D and related metabolic and cardiovascular diseases.

## Methods

### Study design and participants

The EPIC-InterAct study is a large-scale prospective study nested in the European Prospective Investigation into Cancer (EPIC) study, facilitating the investigation of genetic and lifestyle factors on the risk of T2D among European populations. A total of 26 research centres located in eight different European countries (France, Italy, Spain, UK, the Netherlands, Germany, Sweden, and Denmark) were included. The study design, sample collection and genotyping have been described in detail previously^5,6^.

**Fig. 1:**
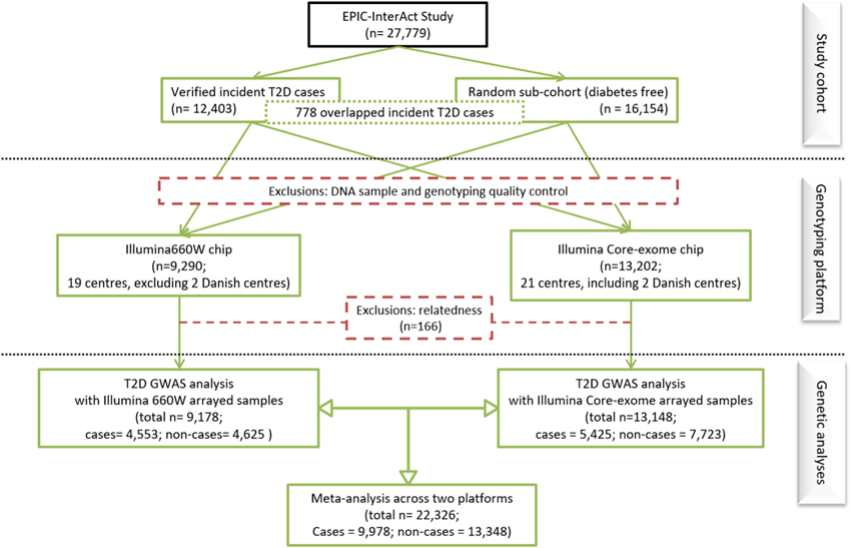
Overview of the EPIC-InterAct study, genotyping and genome-wide association meta-analysis for T2D in 22,326 participants.

In brief, the EPIC-InterAct study adopted a nested case-cohort design. A total of 340,234 participants with stored blood and information reported on diabetes status from the wider EPIC study were followed up for 3.99 million person-years. During the follow-up, researchers from participating study centres ascertained and verified 12,403 incident cases of T2D through self-reported history of T2D, doctor diagnosed T2D and diabetes medication use, linkage to primary care registers, secondary care registers, medication use (pharmacy/ drug registers), hospital admissions and mortality data or local and national diabetes and pharmaceutical registers^5^. To select a representative sub-cohort, a total of 16,835 participants were randomly selected at baseline with numbers proportional to the number of participants in each participating centre. Participants with prevalent (n = 548), unknown (n = 129) and post-censoring diabetes status (n = 4) were excluded, with a total of 16,154 diabetes-free individuals remaining in the EPIC-InterAct sub-cohort (Fig. 1).

### DNA samples and genotyping platforms

Blood samples were collected at recruitment and stored in liquid nitrogen at the International Agency for Research into Cancer (IARC) in Lyon, France, or in local biorepositories except for Umeå where −80 °C freezers were used. DNA was extracted and quantified, with details of sample handling described elsewhere^5,7^.

Available EPIC-InterAct DNA samples were genotyped using two genotyping platforms. A total of 10,023 EPIC-InterAct participants were randomly selected for genome-wide genotyping using the Illumina 660W-Quad BeadChip (Illumina, Inc., San Diego, California) at the Wellcome Trust Sanger Institute with the number of individuals selected per centre being proportional to the percentage of total cases in that centre, except the Danish participants who did not have available DNA samples at the time^7^. Samples were excluded if they had a low call rate (<95.4%), a lack of concordance with previous genotyping results, a mismatch between self-reported sex and the sex inferred from genetic data (X chromosome heterozygosity) or missing data, or they were autosomal heterozygosity outliers, overall array intensity outliers, ethnic outliers (non-European ancestry) or duplicate samples. Related individuals in the Illumina 660 W genotyping array group were identified based on an identity by descent (IBD) pi-hat threshold of 0.1875 (mid-point between second-degree (0.25) and third-degree (0.125) relatives), and those with the largest number of relatives or the lowest call rate were removed preferentially. A total of 9,290 samples genotyped on the Illumina 600 W array passed initial sample quality control (QC).

A total 13,474 individuals from the remaining of EPIC-InterAct samples (including the Danish samples) were genotyped using the Illumina core-exome 12v1 and 24v1 arrays at Cambridge Genomic Services in the Department of Pathology at the University of Cambridge. The two core-exome arrays are very similar; hence the genotype data were merged for further analyses. Following comparable QC procedures as above, a total of 13,202 samples genotyped using the core-exome arrays passed initial sample QC.

Following initial sample QC, an additional 166 participants who had relatives (IBD pi-hat threshold of 0.1875) across the different genotyping arrays (Illumina 660 W vs Illumina core-exome) were excluded, and a total of 22,326 individuals were included in the downstream genetic analyses (Fig. 1; Table 1).

**Table 1:** Sample size of the EPIC-InterAct T2D GWAS analysis by diabetes outcome status and genotyping array.

Diabetes Outcome	Genotyping Array		Total
	Ill660W	Core-exome	
**non-case**	4,625	7,723	12,348
**case**	4,553	5,425	9,978
**Total**	**9,178**	**13,148**	**22,326**

### Genotype imputation

Prior to imputation, single nucleotide polymorphisms (SNPs) were removed if they had Hardy Weinberg p-value < 10^−6^ or were not found in the Haplotype Reference Consortium (HRC) reference panel version 1.0^8^, were A/T or G/C with minor allele frequency (MAF) >0.4, had an allele frequency difference >0.2 with the reference panel, or were short insertion-deletion mutations (indels). A total of 553,115 and 366,044 SNPs passed pre-imputation SNP QC in the Illumina 660W-Quad BeadChip and combined Illumina core-exome arrays, respectively. Imputation was performed using the HRC reference panel and IMPUTE v2.3.2 software^9^. Monomorphic and singleton SNPs and those with imputation quality (info) <0.3 were excluded prior to genetic analyses.

### Genome-wide association meta-analysis

For genome-wide association analysis of T2D, all 22,326 included individuals in the EPIC-InterAct study were of European ancestry, including 9,978 type 2 diabetes cases (including 616 cases from the sub-cohort) and 12,348 non-cases from the sub-cohort, among whom 9,178 participants were genotyped on the Illumina 660 W array and 13,148 using the core-exome array (Fig. 1; Table 1). The mean follow-up time for the EPIC-InterAct cases included in the analyses was 6.8 years (standard deviation (s.d.) =3.3 years), and 12.2 years (s.d. =2.0 years) for the sub-cohort.

We used logistic regression to test genome-wide associations with T2D, rather than Prentice- weighted Cox regression that takes into account the case-cohort design of EPIC-InterAct. Logistic regression was chosen both for computational efficiency and because it has been shown to have greater power than Prentice-weighted Cox regression to detect SNP-disease associations^10^. All T2D incident cases including those from the sub-cohort were coded as ‘1’, and non-cases from the sub-cohort were coded as ‘0’. To estimate the association between T2D and each genetic variant, we performed logistic regression under an additive genetic model, adjusting for age, sex, study centre and the first four genetic principal components to account for population structure using QUICKTEST Version 0.98^11^. Dummy variables for each study centre (combining the six centres in France due to the small sample size in each French centre) were included in the model to account for the differences between participants from each country and the potential confounding by larger scale relatedness between participants from each study centre. Genome-wide analyses were performed separately for each genotyping array and combined using an inverse variance weighted fixed-effect meta-analysis in METAL^12^. The final meta-analysis had an effective sample size^12^ of up to 21,924.

### Ethics statement

The EPIC-InterAct study was approved by the local ethics committee in the participating countries and the Internal Review Board of the International Agency for Research on Cancer. All participants gave written informed consent. The study was coordinated by the Medical Research Council Epidemiology Unit at the University of Cambridge.

## Data Records

Genome-wide association summary statistics from the meta-analysis of T2D in the EPIC-InterAct study and Cox-regression analysis results for the 370 top T2D SNPs from the recently published DIAMANTE study^2^ are available to download from the Dryad Digital Repository (10.5061/dryad.qnk98sfcg)^13^.

The genome-wide summary statistics are in tab-delimited TXT format, including rsID (based on the HRC reference panel), chromosome, position (using the reference genome GRCh37 (hg19)), effect allele, other allele, frequency of effect allele, effect estimate, standard error of the effect estimate, p-value, assessment of heterogeneity across the two genotyping arrays, total sample size and effective sample size for the SNP.

The Cox-regression analysis results are in tab-delimited TXT format, including MarkerName (hg19), rsID (based on the HRC reference panel), chromosome, position (using the reference genome GRCh37 (hg19)), effect allele, other allele, frequency of effect allele, beta, standard error of beta, hazard ratio (HR), lower-bound of 95% confidence interval (CI) of HR, upper-bound of 95% confidence interval (CI) of HR, p-value, imputation quality, total sample size.

Alternatively, the genome-wide summary statistics data is also available in NHGRI-EBI’s GWAS Catalog with accession ID GCST90006934^14^. It can be downloaded via the following ftp link: ftp://ftp.ebi.ac.uk/pub/databases/gwas/summary_statistics/GCST90006934.

In addition, access to individual-level EPIC data is available through the International Agency for Research on Cancer (IARC): https://epic.iarc.fr/access/, where there is a controlled-access repository. A clear and open access request mechanism and data use agreement is in place.

## Technical Validation

For the meta-analysis, only SNPs with minor allele frequency (MAF) > 0.5%, imputation information score > 0.4, Hardy-Weinberg Equilibrium p-value > 1 × 10^−6^ and association effect standard error < 10 from each genotyping platform were included. After the meta-analysis, 31 SNPs with heterogeneity p-value < 1 × 10^−5^ were excluded. A total of 8,924,492 SNPs remained in the shared meta-analysis results. The numbers of genetic variants in each MAF bin are shown in Table 2.

**Table 2:** Number of SNPs in each minor allele frequency (MAF) bin in the final EPIC-InterAct T2D GWAS meta-analysis result after quality control.

MAF bin	(0.005,0.01]	(0.01, 0.05]	(0.05, 0.1]	(0.1,0.2]	(0.2, 0.3]	(0.3,0.4]	(0.4,0.5]
N of SNP^a^	1,201,837	2,287,670	1,117,493	1,429,429	1,074,208	937,594	876,261

^a^SNPs with meta-analysis heterogeneity p value < 10^−5^, imputation info <0.4 and minor allele count <  = 10 were excluded.

**Fig. 2:**
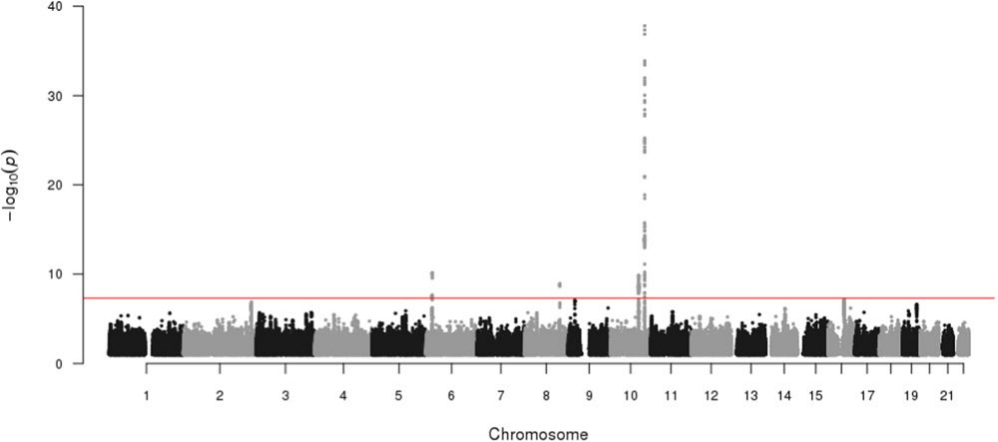
Manhattan plot of genome-wide association meta-analysis for T2D in 22,326 participants from the EPIC-InterAct study. The x-axis is chromosome position (Build 37), and the y-axis is the negative log_10_ p-value (−log_10_(p)) of the association between each genetic variant and T2D. Points represent a genetic variant included in the study (only SNPs with a p-value < 0.1 are illustrated in the plot). The red horizontal line represents the genome-wide significance threshold p-value of 5 × 10^−8^.

**Fig. 3:**
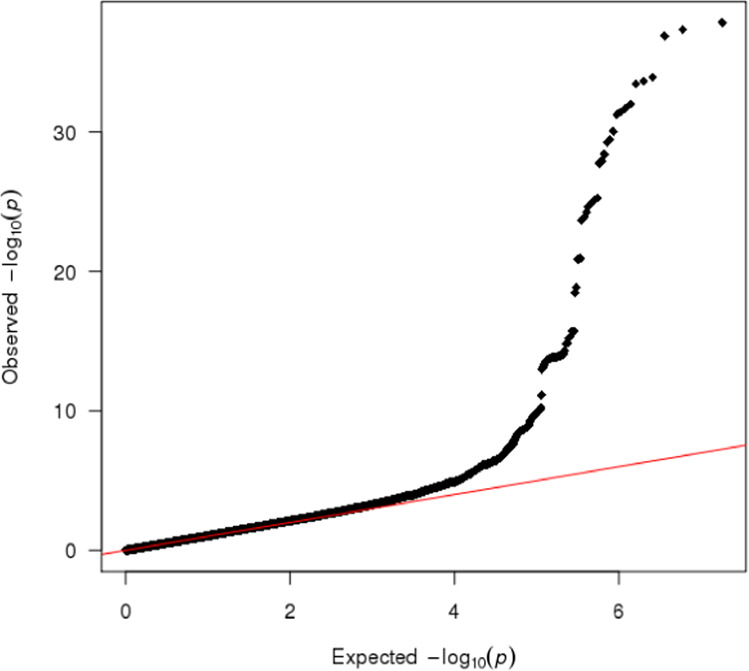
Quantile-Quantile plot of the T2D genome-wide association meta-analysis results in the EPIC-InterAct study.

The Manhattan plot is shown in Fig. 2. The quantile-quantile plot (Fig. 3) showed no evidence of inflation from confounding or other biases, supported by the LD score regression^15^ intercept, which was very close to 1 (1.0054); therefore, no genomic control correction was performed. As a positive control, the top independent genome-wide significant signal from the meta-analysis was the well-established *TCF7L2* variant rs7903146^16^ (p = 1.30 × 10^−38^).

**Fig. 4:**
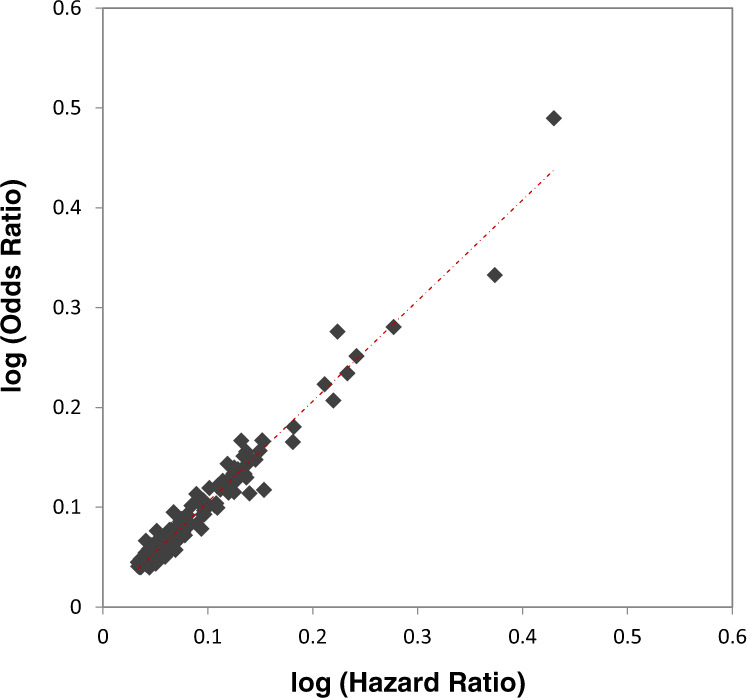
Log(hazard ratios) from the Prentice-weighted Cox regression model and log(odds ratios) from the logistic model for established genetic variants from a previous meta-analysis^2^ with p < 0.05 in the EPIC-InterAct T2D GWAS summary statistics (n = 175).

Because logistic regression may potentially yield inflated effect estimates when applied in a case-cohort study^10^, we compared the strength of associations from the GWAS meta-analysis (logistic regression) and Prentice-weighted Cox-regression analyses adjusting for sex, study centre and first four principal components with age as the underlying time-scale variable for established T2D genetic variants. A total of 370 SNPs from the recently published DIAMANTE study^2^ are available in our HRC imputed EPIC-InterAct genotype data. Among these, 175 SNPs with p-value < 0.05 in the EPIC-InterAct meta-analysis results were included in the comparison. The Pearson correlation coefficient between the log of hazard ratios from the Cox-regression model and the log of odds ratios from logistic regression models was 0.98 (p = 3.1 × 10^−126^) (Fig. 4), showing the effects are highly comparable.

## Data Availability

IMPUTE v2.3.2: https://mathgen.stats.ox.ac.uk/impute/impute_v2.html QUICKTEST Version 0.98: http://toby.freeshell.org/software/quicktest.shtml METAL: https://genome.sph.umich.edu/wiki/METAL All other analyses, including the Prentice-weighted Cox-regression analyses, were performed using R 3.4.2^17^.
